# Efficient and unbiased metagenomic recovery of RNA virus genomes from human plasma samples

**DOI:** 10.1038/s41598-017-02239-5

**Published:** 2017-06-23

**Authors:** Carmen F. Manso, David F. Bibby, Jean L. Mbisa

**Affiliations:** Antiviral Unit, Virus Reference Department, National Infection Service, Public Health England, Colindale, London, NW9 5EQ United Kingdom

## Abstract

RNA viruses cause significant human pathology and are responsible for the majority of emerging zoonoses. Mainstream diagnostic assays are challenged by their intrinsic diversity, leading to false negatives and incomplete characterisation. New sequencing techniques are expanding our ability to agnostically interrogate nucleic acids within diverse sample types, but in the clinical setting are limited by overwhelming host material and ultra-low target frequency. Through selective host RNA depletion and compensatory protocol adjustments for ultra-low RNA inputs, we are able to detect three major blood-borne RNA viruses – HIV, HCV and HEV. We recovered complete genomes and up to 43% of the genome from samples with viral loads of 10^4^ and 10^3^ IU/ml respectively. Additionally, we demonstrated the utility of this method in detecting and characterising members of diverse RNA virus families within a human plasma background, some present at very low levels. By applying this method to a patient sample series, we have simultaneously determined the full genome of both a novel subtype of HCV genotype 6, and a co-infecting human pegivirus. This method builds upon earlier RNA metagenomic techniques and can play an important role in the surveillance and diagnostics of blood-borne viruses.

## Introduction

Single-stranded RNA viruses exhibit exceptional genetic diversity due to low fidelity replication mechanisms^1, 2^. As a group, they constitute the major source of emerging infections in humans such as Ebola, chikungunya, Zika, West Nile virus and Middle East respiratory syndrome-related coronavirus^3–7^. With new human RNA viruses being discovered each year^8^, the serological and nucleic acid amplification techniques that have dominated virus diagnostics for many years are becoming increasingly unable to respond to the ever-expanding range of pathogens.

In parallel, there is a substantial global burden of long-established blood-borne RNA viruses of which the most prevalent are human immunodeficiency virus (HIV) and hepatitis C virus (HCV), infecting over 38 million and at least 100 million people worldwide, respectively^9, 10^. In developed countries, blood and transplant products are routinely screened for these and other viruses. However, even within these two relatively well-characterised virus species, new genotypic divisions and recombinant variants are being discovered^11–13^, and questions have been raised regarding the reliability of commercial assays in detecting new strains^14–16^.

Isolated from humans and several animal hosts, including pigs, Hepatitis E virus (HEV) has, over the course of the last decade, become the leading cause worldwide of acute viral hepatitis. With an estimated 56,600 deaths annually, it is a prime example of an emerging zoonosis with significant blood safety implications, for which conventional serological screening methods seem poorly developed^17, 18^.

New avenues in diagnostic assay development have been opened up by the advent of Next Generation Sequencing (NGS) technologies. Metagenomics – the direct genetic analysis of genomes contained within a sample – represents one such possibility; instead of targeting specific genomic regions of predetermined targets, short DNA sequences (‘reads’) are generated that derive from the full range of genomic material present in the sample^19^. A number of groups have attempted to detect and characterise RNA viruses of clinical relevance directly from sample material, but the low relative abundance of viral genomic material within clinical samples when compared to host-derived nucleic acid species limits their potential utility in diagnostic virology. In the absence of significant host depletion, genome coverage and depths are often low even at high viral copy numbers^20–23^.

Several reports detail methods for partitioning viral and host material through physicochemical purification of virus particles using various combinations of filtration, differential centrifugation, precipitation and extra-virion nuclease digestion prior to extraction, but these also suffer from low virus read frequency and consequent low depth of coverage of partial genomes^24–27^.

The most frequent alternative method is to selectively eliminate host nucleic acids, specifically ribosomal RNA species (rRNA), as they constitute approximately 80% of total cellular RNA. ‘VIDISCA’ is one such approach, where viral nucleic acids are preferentially amplified through the use of non-random hexamers that do not complement human rRNA sequences. In combination with NGS, partial genomes of novel viruses have been detected by this method^28, 29^. As with non-depleting methods, extensive further work is needed to fully characterise detected viruses, and when applied to diagnostic virology, the read depths and genome coverages remain low even at high viral loads^30–33^. Most groups selectively deplete rRNA by hybridising rRNA-specific DNA ‘scissor probes’ to the extracted nucleic acids, and digesting the rRNA/DNA duplexes with RNAse H^34, 35^. This approach has been exploited to good effect with Lassa and Ebola viruses^36^.

The extremely low amounts of RNA surviving host depletion (often in the picogram range) present a significant challenge to RNA library preparation methods, which typically require at least 100 ng of starting material^37^. A Φ29-based Multiple Displacement Amplification (MDA) system has been successful in generating whole genomes of HIV from low copy number samples. However, these were prepared by diluting high titre clinical HIV samples in PBS, such that the impact of high host background was mitigated^38^, and in general, MDA displays target amplification biases that limits its potential in metagenomics to detection and identification rather than whole genome reconstruction^39–41^.

In this study, we have established a sequence-independent RNA library preparation method suitable for the detection and characterization of blood-borne RNA viruses. The method is focused on increasing the relative abundance of viral RNA within the sample, during and after the RNA extraction process, with a specialised library preparation step able to process ultra-low RNA inputs. The RNA enrichment steps enable recovery of a higher proportion of reads of viral origin, constituting a major advance in making virus genome assembly less challenging, leading to notable improvements in sequencing coverages and depths.

The protocol was tested in complex host-enriched samples containing HCV, HIV and HEV, and complete genomes were recovered from the equivalent of 2,000 IU/ml. A mixed virus panel comprising 18 different human RNA viruses, diverse in terms of genomic and structural characteristics, was analysed in order to evaluate the capability of the protocol to detect potential new or emerging viruses present in a plasma sample. By applying the method to a series of samples taken from an HCV-infected patient, we have demonstrated the utility of this technique in fully characterising the strain of HCV together with the complete genome of a previously undetected human pegivirus virus. Further investigation demonstrated that this patient’s HCV virus constituted a new subtype within genotype 6. A second manuscript detailing this clinical case is in preparation.

## Methods

### Ethics statement

All experiments were performed in accordance with the ‘Guidance on Conducting Research in Public Health England’ (Version 3, October 2015; Document code RD001A). This study only involved the use of archived, residual samples that were sent to the National Reference Laboratory for routine diagnosis and sequence characterization with consent for leftover sample to be used in other assays. The samples were anonymized by removal of any patient identifiable information and assignment of a non-specific project number prior to genetic characterization.

### Sample sets

#### Blood-Borne Virus (BBV) Panel

A complex host-enriched sample was prepared by diluting in negative human plasma (NHP, negative for each HIV, HCV, and HEV) stored plasmas from four samples previously characterized by routine diagnostic testing to contain HCV (x2), HIV and HEV (see Table 1 for details). NHP was obtained by centrifuging negative human blood for 10 minutes at 500× *g* to remove cell debris. The final concentration of each virus in the primary panel sample was 10^6^ IU/ml (copies/ml for HIV – implied by IU henceforth for convenience), and three serial tenfold dilutions in NHP were prepared from this stock.

**Table 1:** Details of the four samples combined to create the Blood Borne Virus Panel.

Sample	Diagnosis	Viral Load	Genotype
1	HCV	2.0 × 10^7^ IU/ml	1a
2	HCV	1.9 × 10^7^ IU/ml	1b
3	HIV	2.3 × 10^7^ copies/ml	B
4	HEV	1.0 × 10^8^ IU/ml	3.2

Viral load quantification for the HEV sample was performed in-house^44^, as was genotyping of the HCV^43^ and HEV^68^ samples. Otherwise, the diagnostic assays were performed by the source laboratory. HCV and HIV viral loads were confirmed using methods from refs 45 and 46 respectively.

#### Virus Multiplex Reference (VMR) Panel

A reagent comprising a suspension in PBS of 18 RNA viruses with different genomic and structural characteristics was provided by the National Institute for Biological Standards and Controls (NIBSC, Potters Bar, UK). Each viral component and its approximate relative concentrations is given in Mee *et al*.^42^. Prior to extraction, the panel was mixed 1:1 with NHP. Duplicate 400 μl extractions were performed (representing 200 μl of the original panel volume), together with a single 200 μl extraction of the original panel suspension.

#### Clinical samples of indeterminate HCV genotype

Four plasma samples collected from a patient between 2014 and 2016 were submitted to Public Health England (PHE) for metagenomic analysis as previous genotyping results had been inconsistent. The most recent such test employed NS5b sequencing^43^, and reported the presence of a virus belonging to genotype 6 but was unable to resolve the subtype with any further precision.

### RNA extraction and quantification

Before extraction, all samples were centrifuged for 10 min at 2,500 × *g* to remove cell debris. Triplicate, duplicate and single extractions were performed on the diluted VMR Panel samples (referred to as ‘VMR Panel A/B/PBS’), the BBV Panel samples (‘10^6-3^-A/B’), and the patient sample series, respectively. A negative control comprising 200 μl of the same plasma used to dilute the panels was also extracted.

The SPLIT RNA extraction kit (Lexogen) was used to extract 200 μl of each sample input, according to the manufacturer’s instructions. Acidic phenol was used to preferentially recover the large RNA fraction, which was eluted in 12 μl of nuclease-free water. RNA eluates were quantified using Qubit RNA HS Assay Kit (Thermo Fisher Scientific), which is accurate for concentrations between 250 pg/μl and 100 ng/μl.

### Depletion of ribosomal RNA and DNA digestion

Ribosomal RNA depletion and DNA digestion was achieved using the RiboErase kit (KAPA Biosystems). As all sample extracts were below the detection limit of the Qubit quantification system, the total RNA input was less than the recommended 100 ng. The manufacturer’s specifications were followed with the exception of using the entire 10 μl of the extract, and after the DNA digestion reaction clean up, eluting the residual RNA was in 10 μl of nuclease-free water.

In the case of the BBV Panel, two of the three extracts of each dilution were treated with the RiboErase kit before RNA library preparation. The third set of extracts remained untreated and was used to monitor the effect of the rRNA depletion and DNA digestion upon the subsequent library preparation and sequence analysis. In the case of the VMR Panel (the two duplicates) and the negative control, rRNA and DNA depletion was performed on all extracts. In the case of the uncharacterized HCV strain, extracts from all four samples were treated with RiboErase. An additional, untreated, extract of sample 4 was included, again to monitor the process.

### RNA library preparation with ultra-low RNA input

Libraries were constructed from 10 μl of extracted RNA or 10 μl of rRNA-depleted DNAse-digested RNA, using the NEBNext Ultra Directional RNA Library Prep Kit (New England Biolabs). As the protocol is designed to use a minimum RNA input of 10 ng, several modifications were made to adapt it to an ultralow RNA input. These are listed in Table 2. Libraries were analysed for size distribution using the High Sensitivity DNA Kit (Agilent) on a 2100 Bioanalyser Instrument, and were quantified using the KAPA SYBR FAST Universal qPCR Kit for Illumina libraries (KAPA Biosystems) on a 7500 Real-Time PCR System (Applied Biosystems).

**Table 2:** Protocol modifications made to the RiboErase and NEBNext Ultra Directional RNA Library Prep kits.

Kit	Step	Manufacturer’s recommendations	Protocol modification
RiboErase	RNA input	0.1–1 µg	<2.5 ng
	RNA elution after DNAse digestion clean up	22 μl of 1X fragment prime and elution buffer	10 μl of nuclease-free water
NEBNext Ultra Directional RNA Library Prep Kit	RNA fragmentation reaction volume	Final volume 10 μl:	Final volume 15 μl:
		5 μl RNA	10 μl RNA
		4 μl of NEB Next 1^st^ strand	4 μl of NEB Next 1^st^ strand
		synthesis reaction buffer	synthesis reaction buffer
		1 μl of random primers	1 μl of random primers
	RNA fragmentation time	94 °C for 15 min (Intact RNA)	94 °C for 1 min
		94 °C for 7–8 min (Degraded RNA)	
	1^st^ strand cDNA synthesis	Final volume 20 μl:	Final volume 20 μl:
		10 μl of fragmentation reaction	15 μl of fragmentation reaction
		0.5 μl of Murine RNAse Inhibitor	0.5 μl of Murine RNAse Inhibitor
		5 μl of Actinomycin D (0.1 µg/ µl)	3.5 μl of nuclease-free water
		1 μl of ProtoScript II RT	1 μl of ProtoScript II RT
	Double strand cDNA purification	DNA eluted in 60 μl of nuclease-free water	DNA eluted in 40 μl of nuclease-free water^*^
	End Repair/A-tailing reaction	Final volume 65 μl:	Final volume 50 μl^*^
		55.5 μl of purified cDNA	40 μl of purified cDNA
		6.5 μl of NEBNext End Repair	5 μl of NEBNext End Repair
		Reaction Buffer	Reaction Buffer
		3 μl of NEBNext End Prep Enzyme Mix	5 μl of NEBNext End Prep Enzyme Mix
	Adaptor ligation	Adaptor concentration of 18 nM	After RiboErase treatment:
			Adaptor concentration 1.4 nM
			No RiboErase treatment:
			Adaptor concentration of 7 nM
	Adaptor ligation reaction purification	First clean up step:	First clean up step:
		1X Agencourt AMPure XP beads	1X Agencourt AMPure XP beads
		Second clean up step:	Second clean up step:
		1X Agencourt AMPure XP beads	0.85X Agencourt AMPure XP beads
	PCR Reaction Purification	1X Agencourt AMPure XP beads	0.85X Agencourt AMPure XP beads

*Adjustments to the cDNA elution volume and the A-tailing reactions follow Batty *et al*.^69^.

### qPCR

To determine the relative abundances of viral inserts, libraries constructed from the BBV Panel were analysed by qPCRs with primers and probes targeting each of the three viral components (Refs 44–46﻿ and Supplementary Table S1). Reactions were performed using the Quantitect Virus Kit (Qiagen) according to the manufacturer’s instructions.

### Sequencing

Libraries labelled with different indexes were diluted to 2 nM and pooled. Sequencing was performed on an Illumina MiSeq instrument using the MiSeq Reagent Kit V2 (300 cycles) (Illumina) according to the manufacturer’s guidelines, with the following minor modifications. The library pools were denatured with 0.2 N sodium hydroxide for 2 minutes rather than 5, diluted in kit reagent HT1 to produce a 20 pM solution and then these were further diluted to 11 pM. Of this library pool dilution, 600 μl were loaded onto the MiSeq cartridge.

### Data analysis

All paired end FASTQ files were processed with Trimmomatic v0.30, removing the Illumina adaptor sequences, then trimming leading and trailing bases with phred scores below 20. Reads were discarded where the length of either trimmed end was below 50 bases.

For the determination of genome sequences of blood-borne viruses, trimmed FASTQ sets were normalised using the normalise-by-median.py script in the Khmer package (k = 31, C = 5)^47^ and submitted to the SPAdes *de novo* assembler^48^ without error-correction, applying the default kmer sizes of 21, 33, and 55. Output contigs that matched each virus were identified with the nhmmer function of the HMMER v3.1b2 package^49^ using hidden Markov models (HMMs) built from alignments of each virus (detailed in Supplementary Table S2). Where necessary, the ends of contigs were trimmed to the whole genome alignment. BWA MEM (v0.7.5a, default parameters)^50^ was used to map the original trimmed FASTQs to the genome sequence, and the SAM files were converted to BAM files using samtools v0.1.19^51^ while discarding reads with either 0 × 04 and/or 0 × 08 flags set (i.e. retaining only fully-mapped paired-end reads). Base frequencies at each nucleotide position within each component virus sequence were obtained from BAM files using QuasiBAM v2.2, an in-house C++ program that tabulates base frequencies at each nucleotide position within a reference and generates consensus sequences based upon user-defined depth and variant percentages^52^.

Mapping of trimmed paired-end FASTQ to one or more virus reference genomes was also performed using BWA MEM 0.7.5a. In each case, two independent mappings were performed, using as a reference the viral sequences, supplemented firstly by the March 2009 ‘GRCh37’ release of the human genome, and secondly by a set of human rRNA sequences (NR_003286.1, NR_003287.1, V00589.1, NR_003285.2, gij251831106:648-1601, and gij251831106:1671-3229, as per Malboeuf *et al*.^38^). The second file was used solely to derive counts for reads mapping rRNA which would otherwise be subsumed into the human genome mapping results. From the filtered SAM files, the numbers of reads mapping to each reference sequence were counted. Counts for each of the constituent sequences of the human genome and rRNA were pooled into a “human” count and an “rRNA” count. QuasiBAM was used to derive nucleotide frequencies from which depth and coverage data were calculated. A minimum depth of 10 was required for inclusion in a derived consensus sequence for the BBV Panel (1 for the VMR Panel).

#### BBV and VMR Panel sequences

The members of the multi-FASTA reference file for the BBV Panel were obtained by submitting FASTQ sets from the rRNA-depleted sample with the highest virus concentrations to the SPAdes-HMMER-mapping approach described in the previous paragraph. VMR Panel references were derived from sequences obtained from GenBank using accession numbers from Mee *et al*.^42^. Additionally, the complete genome sequence of a human pegivirus (HPgV) present in the plasma diluent was discovered in the SPAdes contigs file. A HMM profile was constructed from an alignment of GenBank sequences (Supplementary Table S2).

#### Sample with uncharacterised HCV

To obtain full-length HCV genomes, each FASTQ set was submitted to the SPAdes-HMMER-mapping process. Where a complete genome was not obtained, HCV-matching contigs were aligned to the full-length genomes using MEGA5^53^. In addition, contigs with length > 5 kb that did not align to the HMM profile were submitted to BLAST^54^ for identification. Following this analysis, an additional pegivirus genome was derived in similar fashion to the HCV genomes, using the same HMM profile as above for the NHP pegivirus. When calculating the read percentages and coverage plots, both sample-derived full-length genome sequences (HCV and HPgV) were used as the reference sequence when mapping that sample’s corresponding trimmed paired-end FASTQs, as well where only incomplete HCV genomes were obtained.

## Results

### Determination of blood-borne virus genomes from complete human plasma

A Blood-Borne Virus (BBV) Panel was prepared, comprising two strains of HCV (genotypes 1a and 1b), and one each of HIV and HEV diluted in NHP to 10^6^ IU/ml. Three tenfold serial dilutions in plasma were made from this original Panel. Ribosomal RNA depletion was performed on two of each set of triplicate extractions prior to all three being subjected to the modified library preparation protocol.

Data from the most concentrated rRNA-depleted samples were used to generate individual virus genome sequences for use in reference mapping. During this data analysis, an unexpected human pegivirus (HPgV) was found and traced to the NHP diluent. The full genome sequence of this HPgV was determined from the 10^3^-B data and included in the mapping references.

Table 3 gives the read counts, genome coverages and median depths for each virus-dilution combination, across each of the three samples per dilution (10^6-3^-untreated/A/B). Each test sample yielded over 800,000 reads with the exception of 10^3^-A, which gave just over 140,000 reads. With the exception of the two 10^6^ samples, in which only a very small volume of NHP was added to the clinical samples, the percentage of reads mapping to the HPgV remained relatively constant at 29–39%. The exception is 10^5^-B, in which the overall viral read percentage was lower than expected, with a corresponding elevation in reads mapping to the human genome suggesting possible incomplete DNAse digestion during the rRNA depletion step (Supplementary Table S3).

**Table 3:** Detailed sequencing data from the BBV Panel.

**Viral Loads**	**Virus**	**Untreated**				**Treated sample A**				**Treated sample B**				**Combined A & B**	
		**Total reads**	**Percent reads**	**Percent coverage**	**Median depth**	**Total reads**	**Percent reads**	**Percent coverage**	**Median depth**	**Total reads**	**Percent reads**	**Percent coverage**	**Median depth**	**Percent coverage**	**Median depth**
10^6^	HCV 1a	2,225	0.13	100	55	97,430	8.84	100	2,611	105,345	8.47	100	2,919	100	5,561
	HCV 1b	1,575	0.10	99.6	39	70,652	6.41	100	1,900	79,299	6.38	100	2,208	100	4,077
	HIV	607	0.04	73.5	15	32,397	2.94	100	909	34,618	2.78	100	913	100	1,824
	HEV	11,342	0.69	99.9	306	479,553	43.51	100	14,973	508,441	40.90	100	16,420	100	31,325
	HPgV	110	0.01	1.8	3	351	0.03	37.0	7	2,964	0.24	95.3	73	98.0	82
	All viruses	15,749	0.95			680,032	61.70			727,703	58.54				
	(inc. HPgV)	15,859	0.96			680,383	61.73			730,667	58.78				
	**Total**	**1,650,567**				**1,102,137**				**1,243,019**					
10^5^	HCV 1a	383	0.04	43.6	9	68,536	4.12	100	1,812	19,307	1.51	100	533	100	2,334
	HCV 1b	194	0.02	12.6	4	38,517	2.31	100	1,072	11,308	0.86	100	302	100	1,384
	HIV	48	0.01	0.5	0	9,647	0.58	100	252	2,782	0.22	97.1	75	100	337
	HEV	884	0.10	87.9	24	151,485	9.10	100	4,830	41,984	3.28	100	1,362	100	6,183
	HPgV	2,314	0.25	98.5	56	486,264	29.20	100	13,384	137,768	10.75	100	3,833	100	17,179
	All viruses	1,509	0.16			268,185	16.10			75,161	5.86				
	(inc. HPgV)	3,823	0.42			754,449	45.30			212,929	16.61				
	**Total**	**921,178**				**1,665,319**				**1,281,912**					
10^4^	HCV 1a	43	0.01	—	0	5,655	0.42	98.1	144	4,923	0.38	99.5	123	99.7	282
	HCV 1b	30	0.01	—	0	3,669	0.27	93.5	95	3,165	0.24	91.2	82	99.4	178
	HIV	8	—	—	0	957	0.07	55.0	12	3,308	0.26	90.2	83	93.5	112
	HEV	148	0.03	4.5	3	14,733	1.09	99.6	456	12,928	1.00	99.7	396	99.7	815
	HPgV	1,421	0.25	95.0	35	505,198	37.35	100	13,949	447,914	34.63	100	12,195	100	26,091
	All viruses	227	0.04			25,014	1.85			24,324	1.88				
	(inc. HPgV)	1,648	0.29			530,212	39.20			472,238	36.51				
	**Total**	**565,967**				**1,352,617**				**1,293,480**					
10^3^	HCV 1a	24	—	—	0	115	0.08	10.4	1	382	0.05	35.1	4	43.5	7
	HCV 1b	19	—	—	0	57	0.04	6.0	0	214	0.03	17.4	2	26.3	3
	HIV	31	—	1.7	0	15	0.01	1.5	0	187	0.02	18.1	0	19.7	0
	HEV	154	0.02	4.3	3	239	0.17	35.0	7	1,094	0.13	72.5	29	83.1	37
	HPgV	1,862	0.22	98.2	47	48,587	34.49	100	1,343	321,986	39.04	100	8,643	100	9,980
	All viruses	228	0.03			426	0.30			1,877	0.23				
	(inc. HPgV)	2,090	0.25			49,013	34.79			323,863	39.26				
	**Total**	**832,831**				**140,883**				**824,444**					

For each of the three samples (Untreated, A and B) at each dilution (10^6^–10^3^), the number and percentage of reads mapping to each virus are given, together with the genome coverages (depth ≥10) and median depths.

The final column gives these last two metrics from the combined data sets of both the A and B samples. Included in the analysis are data for the HPgV discovered in the sample diluent.

With increasing dilution, the total viral read percentages (excluding HPgV) decline from over 60% to 0.23%. Complete and near-complete genome coverages with depths greater than 10 were achieved at 10^6^ and 10^5^ IU/ml for all four viruses. A few short regions in HIV had low coverages (<10) with 10^5^-B, reflecting the reduced overall viral reads in this dataset, but at a minimum depth of 1, 99.6% coverage was achieved with this sample, with only a 32-base sequence in Pol having no coverage. At 10^4^ IU/ml, HCV 1a and HEV continued to give 98.1–99.7% coverage with median depths over 120. HCV 1b and HIV gave 91.2–93.5% coverage (82–95 median depth) and 55.0–90.2% coverage (12–83 median depth) respectively, and in 10^3^-B, despite only 0.23% of all reads mapping to the four viruses collectively, genome coverages of 18.1–72.5% were achieved, with median depths up to 29.

**Figure 1:**
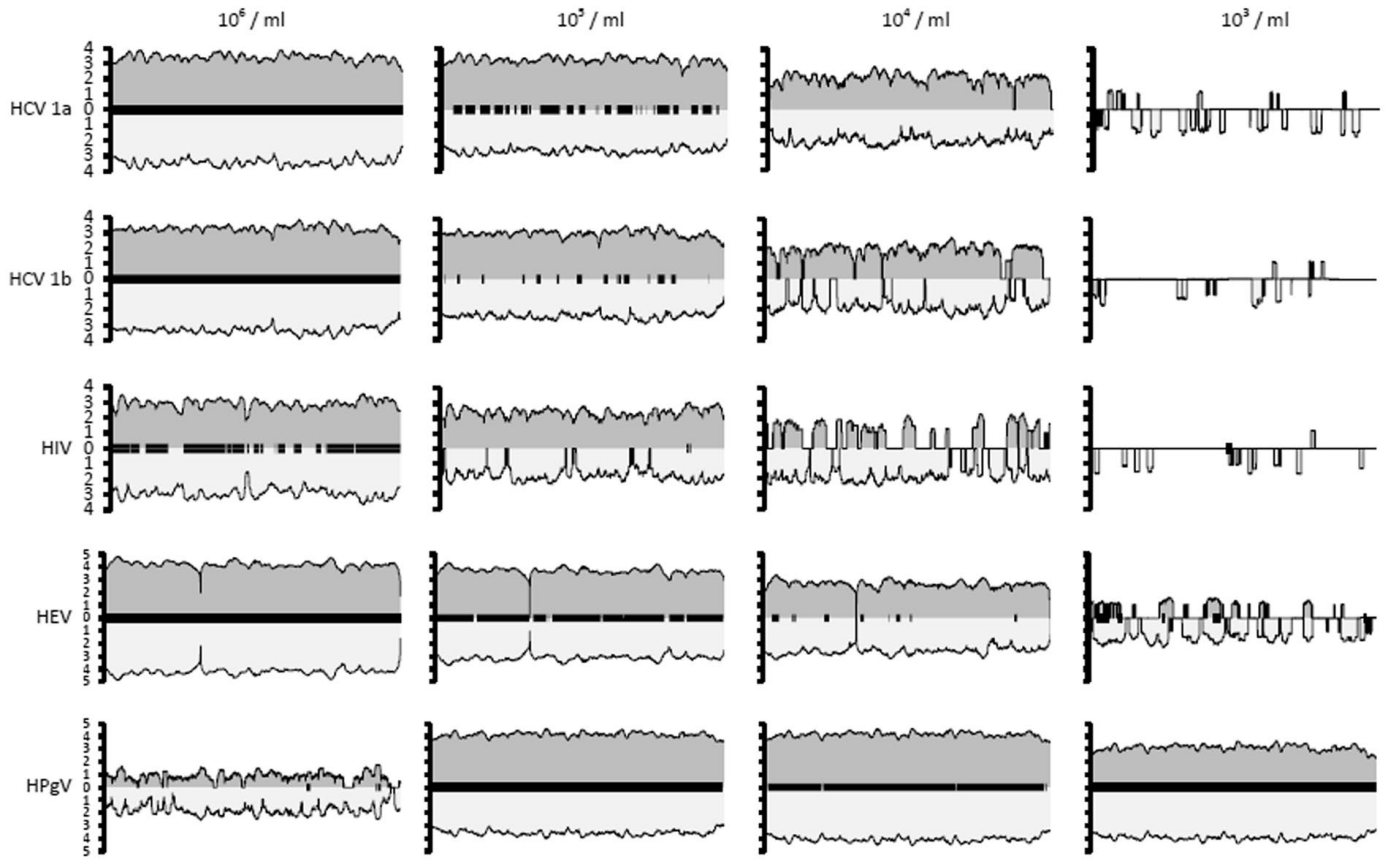
Coverage plots of the Blood Borne Virus Panel samples. The x-axes represent scaled virus genomes, and log_10_ coverages are given on the y-axes. ‘A’ samples are plotted above the axis, ‘B’ samples below. Solid and broken black bars along the x-axes represent where virus coverage of the corresponding ‘Untreated’ sample is ≥10.

Figure 1 illustrates coverages and depths across target genome at each dilution, showing even distributions of reads across all four target genomes and HPgV. Pooling duplicates consistently improved coverages (final column, Table 3). This is most clearly seen at the lower viral loads, where at 10^4^ IU/ml, three of the four viruses achieve combined coverages of >99.4% each, and 93.5% in HIV. At 10^3 ^IU/ml, the combined coverages for the four viruses are effectively what would be expected were the individual coverages independent, i.e. cov_AUB_ ≈ 1 − [(1 − cov_A_)(1 − cov_B_)].

### Depletion of rRNA substantially enhances the recovery of blood-borne virus sequences

The percentage of reads mapping to RNA virus genomes in the rRNA-depleted BBV Panel samples was between 40 and 150-fold higher than in corresponding untreated controls. Individual target virus ratios decreased as they became more dilute, from over 100-fold for HCV in 10^5^-A to 2.9-fold for HIV at the lowest dilution. Concomitantly, the ratio for HPgV rose markedly, from 4.8-fold in 10^6^-A to 175 in 10^3^-B, reflecting an effectively constant viral load against decreasing quantities of Panel viruses (Table 3 and Fig. 1). Genome coverage and median depth values were also much higher in the treated samples than untreated comparators. At the two highest virus concentrations, median depths were between 47- and 274-fold higher in the treated versus the untreated samples. Only short fragments of HEV were recovered from the untreated 10^4^ dilution, and almost no HIV or HCV sequences. By contrast, near complete genomes from all four target viruses were recovered from the treated comparators, with median depths of between 83 and 457 (as noted above, HIV in 10^4^-A was an exception at 54.0% coverage and a median depth of 13).

### Recovery of partial and complete genomes of diverse virus types from human plasma

The ability of our method to recover genome sequences from a range of RNA viruses in the context of human plasma was evaluated using a Virus Multiplex Reference (VMR) Panel, putatively containing 25 genomically and physicochemically diverse viruses. Two plasma-diluted panels and one PBS-diluted panel were tested (Table 4). No reads from either of the three samples mapped to either of the two norovirus genomes, coronavirus 229E or influenza B virus. By the panel distributor’s qPCR^42^, the Threshold Cycle (C_t_) of the coronavirus was >36 and the other three were not detected, hence these four targets were excluded from further analysis. Notwithstanding influenza virus A H3N2 and parainfluenza virus type 3 also not being detected by the qPCR, we recovered reads from both, with genome coverages ranging from 2.7% to 21.6%. Almost no reads belonging to the panel’s DNA viruses were found.

**Table 4:** Detailed sequencing data from the VMR Panel.

**Virus**	**Ct (ref**. 42)	VMR Panel PBS				VMR Panel A				VMR Panel B				A & B
		Total reads	Percent reads	Percent coverage	Median depth	Total reads	Percent reads	Percent coverage	Median depth	Total Reads	Percent reads	Percent coverage	Median depth	Percent coverage
Rotavirus A	24.49	366,740	12.84	69.7	146	84,532	4.27	70.9	25	91,894	4.84	69.0	39	74.3
Parechovirus	29.35	1,559,037	54.58	99.6	49,452	723,168	36.55	99.7	21,649	713,704	37.62	99.8	21,712	99.8
Astrovirus	30.53	9,746	0.34	98.8	319	3,814	0.19	99.6	145	4,449	0.23	96.9	166	99.9
Coxsackie-virus B4	30.72	14,591	0.51	75.8	346	7,777	0.39	78.7	171	7,220	0.38	81.1	183	84.2
Rhinovirus	31.16	5,440	0.19	92.3	215	1,756	0.09	97.9	60	1,825	0.10	96.3	54	98.1
Parainfluenza Virus Type 4	31.83	251	0.01	3.7	0	395	0.02	56.6	1	187	0.01	24.4	0	63.5
Metapneumo-virus	31.86	548	0.02	13.3	0	164	0.01	40.7	0	127	0.01	23.2	0	51.4
Influenza A H1N1	32.02	4	<0.01	4.2	0	40	<0.01	6.8	0	35	<0.01	1.8	0	7.3
Parainfluenza Virus Type 2	33.87	1,316	0.05	20.3	0	47	<0.01	10.0	0	1,728	0.09	88.5	22	88.5
Sapovirus	33.37	436	0.02	8.5	0	56	<0.01	6.6	0	62	<0.01	11.4	0	14.7
Respiratory Syncytial Virus	34.33	86	<0.01	2.4	0	24	<0.01	3.0	0	0	—	—	—	3.0
Parainfluenza Virus Type 1	34.43	468	0.02	11.0	0	141	0.01	34.8	0	189	0.01	26.6	0	49.8
Parainfluenza Virus Type 3	ND	31	<0.01	3.9	0	11	<0.01	2.7	0	12	<0.01	3.2	0	6.0
Influenza A H3N2	ND	175	0.01	5.2	0	162	0.01	28.6	0	86	<0.01	11.9	0	33.0
All VMR viruses	1,958,869	68.58			822,087	41.55			821,533	43.31				
NHP-HPgV					350,242	17.70			317,547	16.74				
Total	2,856,500				1,978,578				1,896,842					

For each of the three samples (PBS, A and B), the number and percentage of reads mapping to each of the 14 viruses are given, genome coverages and median depths.

The final column gives these last two metrics from the combined data sets of both the A and B samples. Included in the analysis of samples A & B are data for the HPgV discovered in the sample diluent.

**Figure 2:**
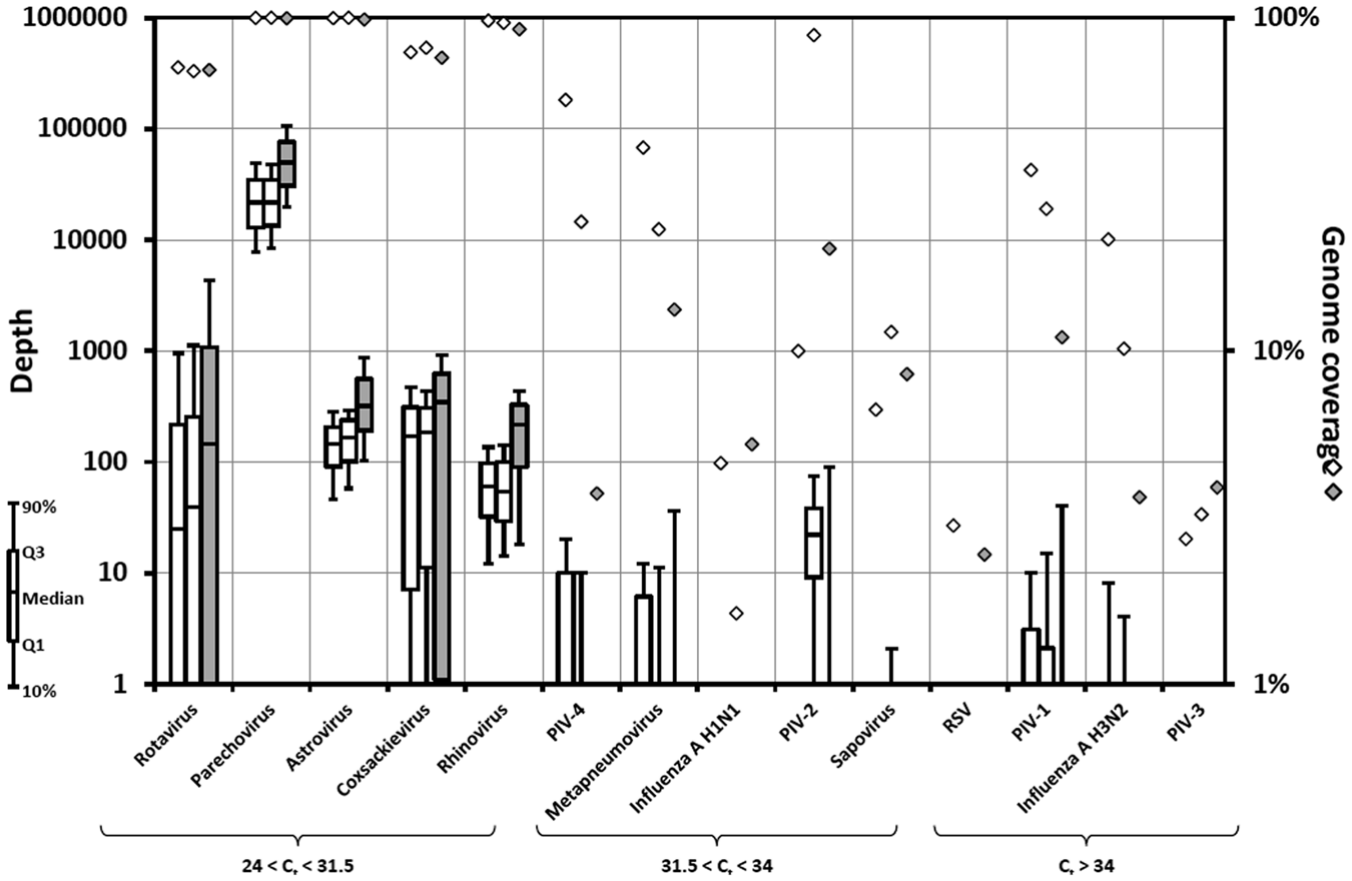
Relationship between viral load, sequencing depth and genome coverage. Analysis of genome coverage (diamonds) and sequencing depth (box-and-whisker plots) for each of the 14 Virus Multiplex Reference Panel viruses analysed. Symbols for the plasma-diluted samples are open and those for the PBS-diluted Panel data are shaded. Viruses have been stratified into three groups by reported C_q_ values^42^.

Sixty-nine percent of all reads obtained from the PBS-diluted panel mapped to VMR Panel genomes, dropping to 41–44% for the plasma-diluted samples, although the distribution of reads between targets was very uneven. Parechovirus and rotavirus accounted for 78.8–87.6% and 10.6–19.5% of all viral reads respectively, with the other viruses collectively accounting for 1.7–1.9%. Depths and genome coverages showed some inverse correlation with the given C_q_ values (Fig. 2).

As with the BBV Panel data, coverage plots of the samples diluted in plasma were largely unbiased, giving pooled genome coverages close to those expected by independent distributions of reads between replicates (Table 4, final column). Rotavirus and coxsackievirus were exceptions, where despite large numbers of mapped reads, almost identical patterns of read coverages and gaps were observed between their replicates, with minimal additive effect. The PBS-diluted sample gave larger read numbers, but their distribution was less even throughout the genomes, resulting in relatively lower coverages.

### Characterisation of a new subtype belonging to HCV genotype 6 and discovery of a second virus in a patient sample series

Four plasma samples from a patient with HCV were used as starting material. All extracts were subjected to RiboErase treatment; a second extract of sample 4 remained untreated for comparison. *De novo* assembly analysis of FASTQ sets from samples 1, 3 and 4 each gave a full-length HCV genome sequence as a single contig. For sample 2, 6 partially-overlapping contigs were obtained, covering 66% of the HCV sequence. Additionally, in all four samples, a single contig was obtained that was determined by BLAST and subsequent HMMER analysis to comprise an HPgV genome.

**Figure 3:**
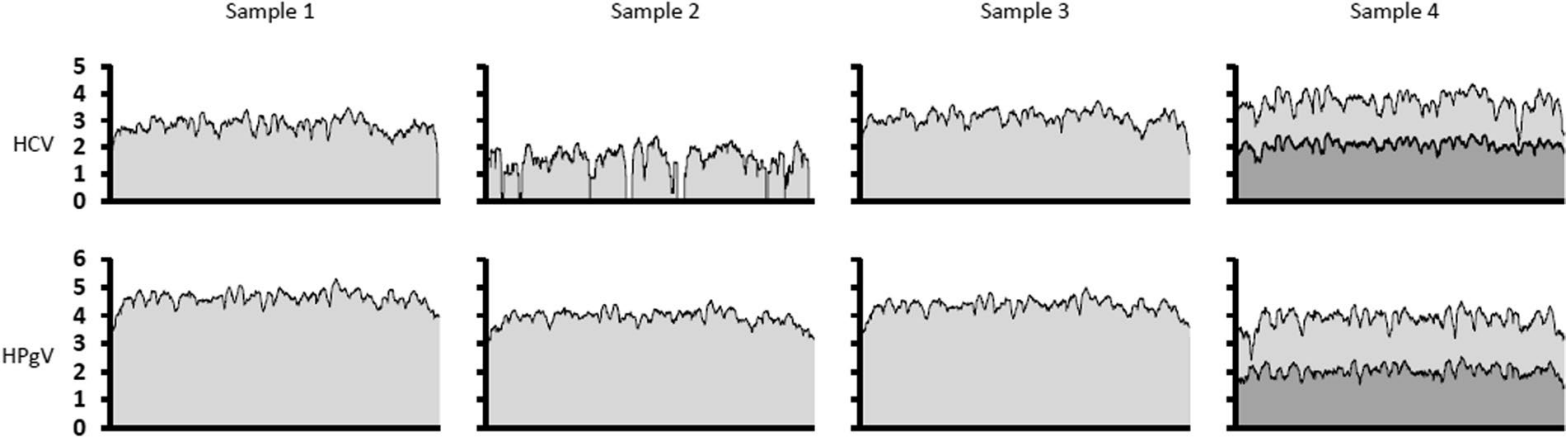
Coverage plots for the HCV and HPgV genomes from the patient sample series. The x-axes represent scaled virus genomes, and log_10_ coverages are given on the y-axes. In the plot for sample 4, the darker plot represents the results of the extract not treated with RiboErase.

The HCV and HPgV full genome sequences were combined in a single file to carry out reference mapping and nucleotide frequency determination on the four sample FASTQ sets (Table 5). Samples 1, 3 and 4 had HCV read percentages ranging from 1.0 to 24.3%, and gave complete genomes with median depths greater than 700. Sample 2 had the lowest viral load (2,000 IU/ml), had 0.3% of reads mapping to HCV giving a genome coverage of 87% at a minimum depth of 10 (96.5% at depth ≥1) and a median depth of 43. Full coverage of the HPgV genome was obtained from all samples, with median depths over 8,700, and read percentages ranging from 34.2 to 63.3%. The depth plots in Fig. 3 again show unbiased and even coverages across both genomes, and the percentages of reads mapping to viral targets was again much higher in the rRNA-depleted sample than in the untreated comparator (61-fold and 85-fold for HCV and HPgV respectively).

**Table 5:** Detailed sequencing data from the patient sample series.

Sample	Viral Load (x10^3^ IU/ml)	Virus	Untreated				RNA/DNA-depleted			
			Total reads	Percent reads	Percent coverage	Median depth	Total reads	Percent reads	Percent coverage	Median depth
1	24	HCV					27,143	1.0	99.4	706
		HPgV					1,640,826	63.0	100	43,724
		Total					2,606,130	64.0		
2	2	HCV					1,854	0.3	87.2	43
		HPgV					332,199	59.7	100	9,719
		Total					556,135	60.1		
3	20	HCV					51,921	2.4	100	1,417
		HPgV					858,790	40.4	100	23,802
		Total					2,123,645	42.9		
4	800	HCV	5,067	0.4	100	133	230,691	24.3	100	5,523
		HPgV	4,131	0.4	100	105	323,266	34.0	100	8,953
		Total	1,129,571	0.8			979,446	58.3		

For each of the four samples 1–4, the number and percentage of reads mapping to both the HCV and HPgV genomes are given, genome coverages (depth ≥10) and median depths. The analysis of sample 4 extracted without host rRNA depletion is in the Untreated column.

Analysis of the HCV sequence showed it to belonging to a new subtype within genotype 6 of which the details are presented in a separate manuscript (in preparation). The HPgV clustered with genotype 1 strains, and is distinct from the NHP strain.

### Analysis of human origin reads and negative control

Libraries from the BBV Panel extractions including the NHP negative control were subjected to virus-specific qPCR for the detection and quantification of HCV, HIV and HEV. All were detectable in the sample libraries, but were undetectable in the RiboErase-treated negative control library (Supplementary Table S4).

**Figure 4:**
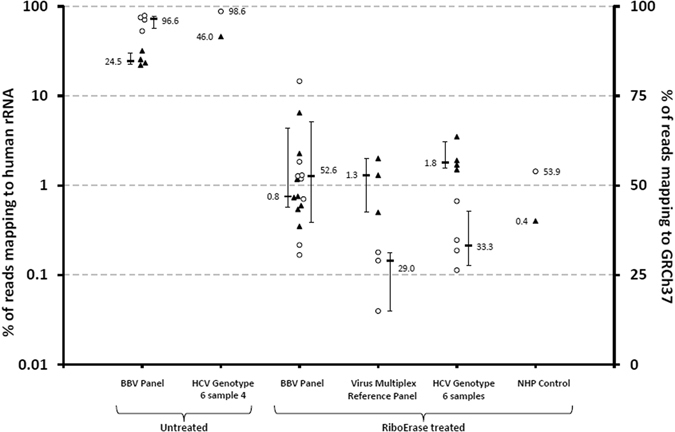
Proportion of total sequencing reads that are of human origin. Across all samples, the percentages of reads mapping to the human genome (open circles) and to ribosomal RNA (closed triangles) is significantly lower in those subjected to RiboErase treatment. Median and interquartile ranges are shown alongside each series.

All samples were mapped against reference sequences that included human genome and human rRNA sequences to evaluate the efficiency of RiboErase treatment. The average ratio of the percentages of reads mapping to rRNA in the untreated versus the treated samples was 32-fold with an approximate halving of the number of reads mapping to the human genome, across all panels (Fig. 4).

With the exception of the expected human pegivirus, mapping of the negative control FASTQ set against the reference sequences of the four BBV Panel viruses, the two pegiviruses, the VMR Panel and the patient HCV gave very low numbers of reads mapped to viral genomes and no consensus sequences could be derived. Further data for this section are found in Supplementary Tables S3 and S5.

## Discussion

In light of the large and ever-increasing number of human RNA virus pathogens, it is perhaps unsurprising that standard serological assays and nucleic acid tests suffer from a lack of sensitivity to diverse variants of target viruses, overlook the presence of new or unexpected viruses, and provide only limited information about those targets they do successfully detect. Hence the three main aims of metagenomic virology are to detect & identify known agents irrespective of their diversity, to discover novel agents of disease, and to obtain complete sequence information of detected viruses. Most existing protocols achieve a maximum of two of these aims, but difficulties in selectively isolating viral RNA species and short read sequences from those of the super-abundant host nucleic acid have limited the utility of metagenomic approaches in diagnostic virology.

This study has addressed these limitations by establishing a novel methodology suitable for the agnostic detection and characterization of blood-borne RNA viruses in plasma samples. By depleting host-derived nucleic acids and making modifications to an existing library preparation protocol to account for ultra-low RNA input quantities, we have been able to reconstruct effectively full-length genomes of HCV, HEV and HIV from plasma samples with viral loads of 10^4^ IU/ml (copies/ml for HIV) and substantial fractions of complete genomes at 10^3^ IU/ml. When applied to a series of clinical samples, we could elucidate simultaneously the full genome sequences of both a novel subtype belonging to HCV genotype 6 and a hitherto-undetected human pegivirus. Additionally, our system was able to recover viral sequences from a panel of diverse RNA viruses diluted in human plasma, with a broad correlation between the genomic coverage and depth metrics and approximate concentration. Although full genomes were not assembled in many cases, the independence of read distribution gave sufficient genome coverage for identification.

The vast majority of RNA molecules in a human plasma sample are host-derived, of which up to 80% comprises the six species of human rRNA. Their presence in our libraries was minimised by two key protocol steps in our modified protocol. Firstly, we selected an extraction method that combined a phenol/chloroform step with a column format (Lexogen SPLIT RNA) which increased the amount of extracted viral RNA by up to one log when compared to other extraction methods (data not shown). Perhaps more importantly, by controlling the final precipitation step, small RNA molecules below 150 nt such as 5 S rRNA and tRNA are excluded from the eluates, as are the majority of molecules of human genomic DNA.

Secondly, we employed DNA probes complementary to human rRNA such that hybridisation and subsequent digestion by RNAse H dramatically reduced their frequency in the finished libraries. Whilst this methodology has been successfully used in the detection and characterisation of two haemorrhagic fever viruses, the frequency of viral reads was often below 1% and an additional hybrid-capture step was employed to elevate read numbers^36^. Methods that do not deplete rRNA generally give poor recovery of viral reads, yielding viral genome fragments that necessitate further work^27, 32, 55, 56^, low read numbers even at viral loads over 10^4^ IU/ml^20–22, 33^, or at best, requiring dilution of both host and virus in PBS in order to recover full HIV genomes at low copy numbers^38^.

The resultant rRNA-depleted sample extracts typically contain quantities of nucleic acid in the low picogram range. Library preparation through hexamer-mediated reverse transcription followed by Multiple Displacement Amplification constitutes an easy and effective means of amplifying very low amounts of DNA^27, 38, 57^, but in several studies (and in the authors’ laboratory), significant amplification biases have been observed, leading to gaps in target genome coverage^39, 58–60^. Consequently, we adopted an approach using a standard RNA library preparation kit, but with substantial modification to compensate for their minimum RNA input requirements of at least 10 ng and optimally 100 ng-1 µg.

We made key changes to the RNA fragmentation and adaptor-ligation steps of the NEBNext Ultra Directional RNA Library Prep Kit protocol. While prior RNA fragmentation with heat and divalent cations improves sequence coverage, over-fragmentation of target genomes leads to the loss of material during the library preparation process^37^. Lower amounts of RNA thus require shorter optimum fragmentation times and we found that 1 minute at 94 °C was optimal in terms of breadth of genome coverage.

Under standard kit conditions, our ultra-low RNA inputs dramatically skewed the ratio of cDNA to adaptor. The resulting adaptor excess led to the preferential amplification of adaptor dimers during the PCR step, and despite increasing cycle number to amplify low RNA inputs, we were generally unable to generate sufficient quantities of target-specific material. Accurate quantification and consequent equimolar pooling of libraries was compromised, as was the MiSeq clustering efficiency. We found that a reduced final adaptor concentration of 1.4 nM was crucial in reducing the amount of adaptor dimers in libraries from rRNA-depleted samples whilst simultaneously extending the PCR cycle number.

In the present study, serial dilutions of the Blood Borne Virus Panel were prepared in negative human plasma, reducing both the absolute quantity and relative frequency of the viral RNA targets while maintaining the complexity of the sample in terms of host nucleic acid, thus mimicking that of a clinical sample. With rRNA depletion, the number and diversity of viral reads was consistently high, with over 35% of all reads mapping to constituent virus genomes. Throughout the three sample series, we obtained relatively high genome coverages of low-frequency viral targets. Co-infections with multiple blood-borne viruses are common^61^, so whilst we speculate that the depths and coverages of target viruses would be greater yet in these samples had it not been for the confounding effect of the unexpected human pegiviruses in both the plasma diluent and the patient sample series, it was reassuring to see the method performed well under such conditions.

In our experiments using negative human plasma as sample diluent, we were able to recover levels of viral genomes comparable to previous work using PBS, both for BBV Panel viruses^38^ and for the VMR Panel^41^, and we were able to recover from a patient sample a large percentage of the genome of a previously uncharacterised subtype of HCV genotype 6 when present at 2 × 10^3 ^IU/ml, a diagnosis not possible using existing genotyping assays. The presence of an undiagnosed pegivirus in this sample further demonstrated the utility of the method in metagenomic analysis of blood-borne virus co-infections where the relative abundances of each virus can be highly variable^22^. Furthermore, in three of the four samples, depths greater than 1,000 were routinely obtained, which are likely to be sufficient to call minority variants for clinical resistance^62^. A full description of the patient series and the new HCV strain are provided in a separate manuscript (in preparation).

Our approach can therefore not only accurately characterise rare or novel variants of existing viruses, but also generates the same level of information regarding unexpected viruses present in the sample. By comparison, VIDISCA^32, 63, 64^ and other random amplification-NGS techniques^30, 31^ have detected novel viruses in diverse clinical samples, but all have required further techniques to achieve full genome sequences.

Together with the VMR Panel results, we were able to recover identifying sequence from both enveloped viruses (HCV, HIV, HEV, influenza, and several paramyxoviruses), and non-enveloped viruses (several enteroviruses, astrovirus, rotavirus, and sapovirus). For the majority of viruses in the VMR Panel, whilst dilution in plasma reduced the total percentage of reads recovered when compared to the panel diluted in PBS, a greater breadth of genome coverage was achieved. In the absence of any host nucleic acid background, it is possible that the PBS extracts had such ultra-low quantities of RNA that despite the adjustments made to the library preparation protocol, the RNA was over-fragmented, leading to a smaller number of genome fragments that were individually amplified to a greater extent than the larger array of fragments surviving the plasma extraction.

In developing a similar approach, Kohl *et al*. were only able to recover a percentage of reads exceeding 6% at a viral load over 10^7^ copies/ml. At an influenza A virus concentration of over 10^5^ copies/ml, this dropped to just 0.5%, and at a reovirus concentration of 10^3^–10^4^ copies/ml, no viral reads were detected^24^. With our method, whole genomes were obtained for those with the highest viral loads, and for minority viral targets, there was a correlation between ostensible quantity and coverage, including for two viruses undetectable by the panel distributor^42^, a result superior to that recently obtained from influenza in clinical respiratory samples^65^. Again, the presence of high quantities of one or more target is likely to have inhibited the representation of the minority species such that if tested individually, superior depths and coverages would seem likely. With further reduction to the fragmentation time, or even its abolition, it may be possible to use this method to reconstruct genomes from old, partially degraded samples such as those recently used to re-evaluate the early HIV epidemic in the Americas^66^.

Our negative control data suggest that the level of contamination is low, with most viral reads therein belonging to the most abundant VMR Panel member. Nelson *et al*.^67^ identified a second source of contamination consisting of incorrect reads from other libraries that were sequenced during the same sequencing run due to TruSeq index misassignment (~0.06% of reads, 0.02% here). Although cross-contamination between samples during the library preparation can be another source of contamination, the qPCR results suggest no BBV Panel genomes were present after library preparation in the negative control sample.

To conclude, by applying the three adaptations of selective large RNA extraction, rRNA depletion-DNAse treatment, and the extensively modified library preparation in combination, NGS data sets can be produced from plasma samples that are rich in RNA virus sequence data. Complex bioinformatic processing has been employed to identify viruses within a metagenomic dataset^7, 25, 26, 32, 64, 65^, but here, only simple bioinformatic processing is needed for detection and identification of known viruses, and by applying only moderately more advanced tools, an agnostic approach to virus detection can be taken, together with characterisation of the full genome even at low viral loads.

## Electronic supplementary material

Supplementary Information
