# Supplementary material for: Efficient and unbiased metagenomic recovery of RNA virus genomes from human plasma samples
Source: Sci Rep. 2017 Jun 23;7:4173. doi: 10.1038/s41598-017-02239-5 (PMC5482852; doi:10.1038/s41598-017-02239-5)
Supplement: Supplementary file 1 — Supplementary Information [file 41598_2017_2239_MOESM1_ESM.pdf]

Efficient and unbiased metagenomic recovery of RNA virus genomes from human plasma samples

Carmen F Manso, David F Bibby, and Jean L Mbisa

Table S1. Primer and probe sequences for quantifying viral representation in libraries.

HCV	Forward primer	ACTGCCTGATAGGGTGCTTG
	Reverse primer	TGGTTTTTCTTTGAGGTTTAGGA
	Probe	FAM-AGTGCCCCGGGAGGTCTCGTAGAC-BHQ
HIV	Forward primer	GGTTTATTACAGGGACAGCAGAGA
	Reverse primer	ACCTGCCATCTGTTTTCCATA
	Probe	FAM-ACTACTGCCCCTTCACCTTTCCAGAG-BHQ
HEV	Forward primer	GGTGGTTTCTGGGGTGAC
	Reverse primer	AGGGGTTGGTTGGATGAA
	Probe	FAM-TGATTCTCAGCCCTTCGC-MGB

Table S2. Sources of the alignments used to generate HMM profiles for use in HMMER. The HCV and HIV alignments are curated and available online for download. For HEV and HPgV, whole genome sequences were obtained from GenBank and aligned using Sequencher v5.0 (Gene Codes). The accession numbers for each virus are given in the table.

Virus	HMM source alignment
HCV	"Alignment of HCV Genotypes and Subtypes" (updated 26th May 2015) obtained from the International Committee on Taxonomy of Viruses
HIV	Los Alamos National Laboratory Web genome alignment<br>www.hiv.lanl.gov/content/sequence/NEWALIGN/align.html
HEV	AB073912, AB074915, AB074917-8, AB074920, AB080575, AB089824, AB091394-5, AB097811-2, AB099347, AB108537, AB189070-1, AB193176-8, AB197673-4, AB200239, AB220971-9, AB222182-4, AB236320, AB246676, AB248520-2, AB253420, AB290312-3, AB291951-68, AB301710, AB362839-43, AB369687-91, AB425830-1, AB437316-8, AB443623-7, AB480825-9, AB521805-6, AB573435, AB591733-4, AB593690, AB602439-41, AB698654, AB720034-5, AB740220-2, AB740232, AB780450-3, AB850879, AB856243, AB909124-5, AF051830, AF076239, AF444002-3, AF455784, AF459438, AJ272108, AP003430, AY115488, AY230202, AY575857-9, AY594199, AY723745, D11092-3, DQ279091, DQ450072, DQ459342, EF077630, EF570133, EU360977, EU366959, EU375463, EU495148, EU676172, EU723512-6, FJ426403-4, FJ457024, FJ527832, FJ610232, FJ653660, FJ705359, FJ763142, FJ906895-6, FJ956757, FJ998008, GU119960-1, GU188851, GU206559, GU361892, GU937805, HM055578, HM439284, HQ389543-4, HQ634346, HQ709170, JF443717-26, JF915746, JN837481, JN906974-6, JQ655733-6, JQ679013-4, JQ740781, JQ768461, JQ953664-6, JQ993308, JX109834, JX121233, JX565469, JX855794, KC163335, KC492825, KC618402-3, KC692453, KF176351, KF736234, KJ013414-5, KJ155502, KJ496143-4, KJ507955-6, KM253769, KP294371, KP698919, KR872414-7, KT447526-8, KT633715, KT727028, KU356182-9, KU670940, KX227751, LC022745, LC037955, LC042232, LC055972-3, LC061267, LC126331-2, LC131066, M94177, NC_001434, X98292
HPgV	AB003288-93, AB008336, AB008342, AB013500-1, AB018667, AB021287, AF006500, AF031827, AF121950, AY196904, AY949771, D87255, D87262, D87708-15, D90600-1, HQ331233, HQ331235, JN127373, NC_001710, U36380, U45966, U63715, U94695

Table S3. Percentages of reads mapping to the human genome and rRNA sequences, for each sample.

Source	Sample	Untreated/PBS sample % rRNA	Untreated/PBS sample % GRCh37	Treated sample A % rRNA	Treated sample A % GRCh37	Treated sample B % rRNA	Treated sample B % GRCh37
BBV Panel	10^6 IU/ml	31.9	93.1	0.3	30.6	1.2	33.3
	10^5 IU/ml	25.5	96.2	0.5	46.2	2.3	79.1
	10^4 IU/ml	23.4	96.9	0.8	52.6	6.5	51.8
	10^3 IU/ml	22.1	97.4	0.7	56.6	1.2	52.8
VMR Panel		2.0	14.9	1.3	31.3	0.5	29.0
Patient series	Sample 1			1.7	31.8		
	Sample 2			3.5	26.3		
	Sample 3			1.9	45.6		
	Sample 4	46.0	98.6	1.5	34.7		
Negative control				0.4	53.9		

Table S4. Analysis of representation in libraries of sequences belonging to BBV Panel viruses. BBV Panel and negative control sample qPCR results are expressed as Ct values

Sample	Virus	10^6 IU/ml	10^5 IU/ml	10^4 IU/ml	10^3 IU/ml
Treated Sample A	HCV	10.8	13.6	17.6	29.7
	HIV	16.3	18.1	21.4	35.3
	HEV	8.5	11.9	16.6	24.0
Treated Sample B	HCV	11.3	13.3	19.9	17.0
	HIV	18.0	21.0	21.3	37.0
	HEV	9.4	12.1	15.3	31.5
Untreated sample	HCV	11.4	13.9	17.1	20.8
	HIV	14.5	18.1	20.1	22.0
	HEV	8.8	12.3	15.3	18.7
Negative control	HCV	Not Detected			
	HIV	Not Detected			
	HEV	Not Detected			

Table S5. Reads mapping to viruses in the negative control sample, including the unexpected HPgV.

Virus	Total reads	% reads
Rotavirus	8	<0.01
Astrovirus	1	<0.01
Parechovirus	125	0.01
PIV-2	3	<0.01
HCV	12	<0.01
HIV	5	<0.01
HEV	-	
HPgV	370,788	36.3
rRNA	3,974	0.4
Human genome	551,014	53.9
Total	1,022,639	
